# A translational platform for prototyping closed-loop neuromodulation systems

**DOI:** 10.3389/fncir.2012.00117

**Published:** 2013-01-22

**Authors:** Pedram Afshar, Ankit Khambhati, Scott Stanslaski, David Carlson, Randy Jensen, Dave Linde, Siddharth Dani, Maciej Lazarewicz, Peng Cong, Jon Giftakis, Paul Stypulkowski, Tim Denison

**Affiliations:** Medtronic NeuromodulationMinneapolis, MN, USA

**Keywords:** automation, closed-loop, neuromodulation, prototyping, hippocampus, seizure

## Abstract

While modulating neural activity through stimulation is an effective treatment for neurological diseases such as Parkinson's disease and essential tremor, an opportunity for improving neuromodulation therapy remains in automatically adjusting therapy to continuously optimize patient outcomes. Practical issues associated with achieving this include the paucity of human data related to disease states, poorly validated estimators of patient state, and unknown dynamic mappings of optimal stimulation parameters based on estimated states. To overcome these challenges, we present an investigational platform including: an implanted sensing and stimulation device to collect data and run automated closed-loop algorithms; an external tool to prototype classifier and control-policy algorithms; and real-time telemetry to update the implanted device firmware and monitor its state. The prototyping system was demonstrated in a chronic large animal model studying hippocampal dynamics. We used the platform to find biomarkers of the observed states and transfer functions of different stimulation amplitudes. Data showed that moderate levels of stimulation suppress hippocampal beta activity, while high levels of stimulation produce seizure-like after-discharge activity. The biomarker and transfer function observations were mapped into classifier and control-policy algorithms, which were downloaded to the implanted device to continuously titrate stimulation amplitude for the desired network effect. The platform is designed to be a flexible prototyping tool and could be used to develop improved mechanistic models and automated closed-loop systems for a variety of neurological disorders.

## Introduction

Neuromodulation devices for deep brain stimulation (DBS) deliver targeted electrical stimulation to treat symptoms of diseases such as Parkinson's disease, essential tremor, and dystonia. To ensure benefit, these therapies require not only accurate placement of the stimulating electrode within neural tissue, but also proper selection of stimulation parameters (e.g., amplitude, pulse width, and frequency). These parameters can be used to mitigate side effects including hemiballism, gait and speech disturbances, and dyskinesias (Limousin et al., 1996, 1998; Hamani et al., 2005; Yu and Neimat, 2008; Bronstein et al., 2011). While many patients benefit from DBS, the parameter selection process is largely heuristic, and reprogramming sessions may be weeks or months apart.

**Figure 1:**
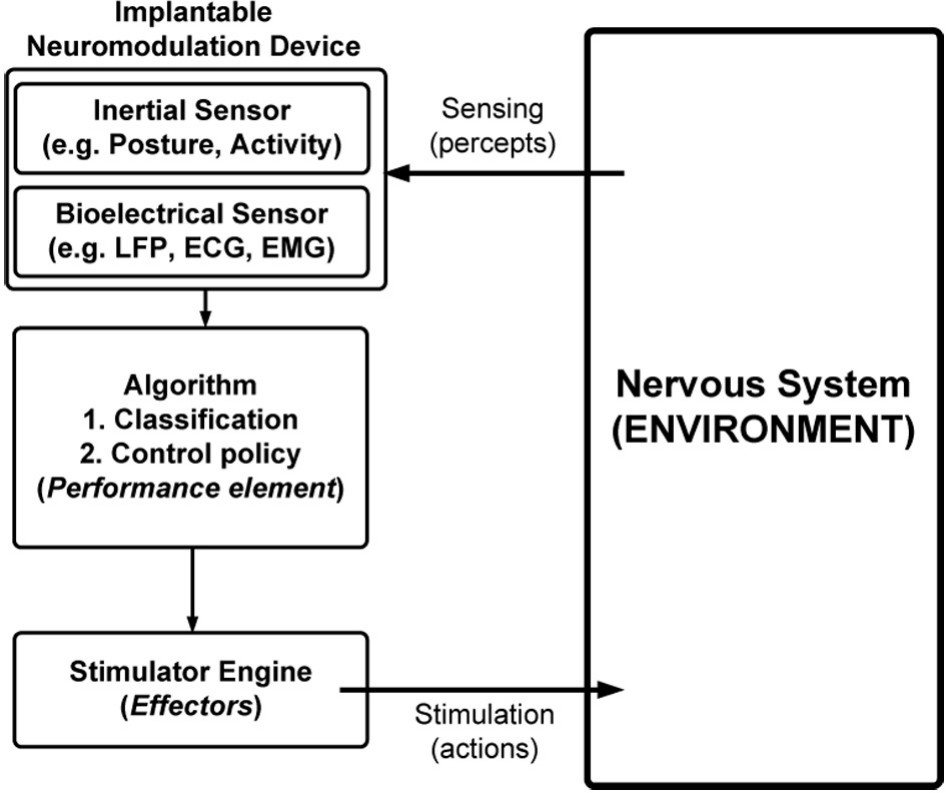
**Simplified model of a closed-loop neuromodulation system**.

Effort has been applied for more than a decade to build automated systems (Figure 1) that use patient state to adjust stimulation parameters, thereby reducing the delay between stimulation updates by many orders of magnitude compared to human intervention. Realizing these systems requires development of sensors to measure patient data and algorithms to translate the data to the appropriate stimulation parameters (Priori et al., 2012). Complexity in the nervous system motivates partitioning the algorithm into two components: one that translates sensor data into estimates of state (i.e., a classifier algorithm) and another that translates the state estimate into a stimulation parameter update (i.e., a control-policy algorithm). In this work, state is left intentionally ambiguous because its meaning depends on the application: examples include seizure versus non-seizure; Parkinson's ON versus OFF; asleep versus awake; or others. Regardless of the application, dividing the algorithm provides the following benefits:

- Matches clinical workflow: clinical practice often separates a patient assessment (“classification”), which translates clinical data into a diagnosis, and a treatment plan (“control policy”), which translates a diagnosis into a therapy. Designing the system to match this separation enables physicians to more easily validate and improve algorithms according to their existing workflow.
- Partitions complexity: algorithms can involve significant computational load, which is difficult for implantable systems due to power constraints (Lee Kyong et al., 2012). Partitioning the algorithm should enable more modular testing and prototyping; this is particularly useful when algorithm components can be externalized to allow greater computational freedom than the implanted device can provide. Once vetted, algorithms with the desired trade-offs between performance, latency, and power consumption can be committed to embedded firmware for untethered operation.

**Figure 2:**
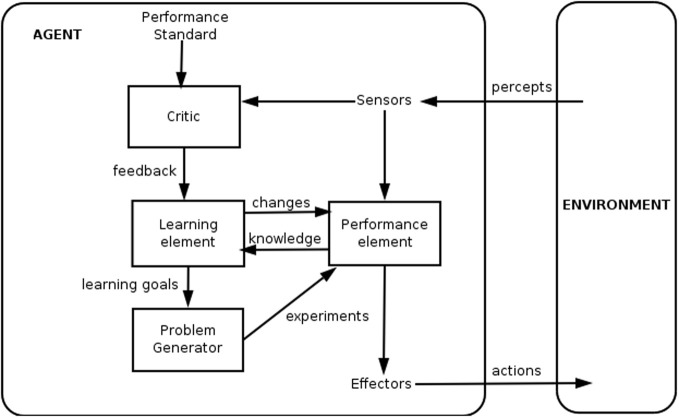
**Generalized framework for a learning agent (reproduced with permission; WikiCommons)**.

The “agent-environment” model from artificial intelligence research is one model for describing the relationship between the physician and the automated neuromodulation system in learning and implementing algorithms (Figure 2). The goal of the agent is to develop a performance element (i.e., algorithm) to model the relationship between environmental percepts and actions taken by effectors. The informed critic (i.e., clinician-researcher) updates the performance element by learning from its input data (sensors) and intermediate processing (knowledge) to develop new problems or hypotheses regarding the algorithm. Iterative testing allows the critic to simultaneously learn about the environment and develop the best performance element to modulate it.

The agent-environment model is suitable for the development of neuromodulation systems for several reasons. The model:

- Includes the physician-researcher's involvement to capture subject behavior to validate the algorithm.
- Describes the role of the performance element not only as a key element of the automated closed-loop.system, but also as the mechanism for the physician-researcher to learn about the nervous system.
- Captures the importance of developing better sensors and effectors to improve the ability to monitor and modulate the nervous system.
- Captures the iterative learning process needed to develop a first-principles understanding of the neurological diseases.
- Leaves the nature of the algorithm open, keeping free the choice of machine-learning techniques (e.g., support vector machine, Kalman filter) and data types (e.g., accelerometer, gyroscope, biopotential).

The translation of automated closed-loop systems has been helped by the development of more sophisticated neural sensors as well as improved understanding of the neural signals that underlie disease. Neurochip-2 (Zanos et al., 2011) and Hermes-D (Miranda et al., 2010) are two examples of technology to measure from the network. Neurochip-2 provides three channels of sensing and stimulation and allows for fast response loop closure to explore concepts like neural plasticity. The Hermes-D system allows for wireless, larger scale measurement (32 channels) of activity, but lacks stimulation capability. Both systems have the advantage of higher bandwidth, which allows for measurement of single unit activity, but draw greater than 1000× more power for operation than a typical DBS implant, giving them longevity of at most a few days between recharges. Moreover, the limited biomarkers and control variables currently known for neurological diseases motivate the development of platform technologies to enable improved first-principles understanding, which may lead to more rapid clinical translation. A critical step in developing this understanding is the ability to provide simultaneous neural recording and therapeutic stimulation, which is lacking in many research tools today. This capability is needed to understand the system transfer function, which we define as the relationship between stimulation and network behavior.

The study of biopotential biomarkers has shown spectral power in local field potentials (LFP) to be a disease-relevant indicator in a variety of settings (Schnitzler and Gross, 2005; Uhlhaas and Singer, 2006). In particular, these signals are useful in studying networks of thalamo-cortical structures and their dynamic inter-relationships, where abnormal neural synchrony is believed to be a hallmark of disease states (Llinas and Ribary, 2001; Siegel et al., 2012). Furthermore, quantified differences in neural synchrony, which can be measured by calculating power (uV/rtHz)^2^ in a particular frequency band (for example, “beta”), have been shown to correlate with symptom severity. For instance, power in the beta band (15–30 Hz) has been found to be related to cardinal Parkinson's symptoms such as bradykinesia and rigidity (Hammond et al., 2007; Eusebio and Brown, 2009; Kühn et al., 2009; Priori et al., 2012). Characteristic changes in power at the theta tremor frequency (Hellwig et al., 2001) and coherent activity in the 6–15 Hz frequency band (Raethjen et al., 2002) have also been found in essential tremor. Synchronization in even lower frequencies (alpha and theta range) has been found in dystonia (Liu et al., 2002; Silberstein et al., 2003; Kühn et al., 2009; Sharott et al., 2008; Singh et al., 2011). Correlations between power in frequency bands as low as alpha (Zumsteg et al., 2006) and as high as 500 Hz (Blanco et al., 2011) have been reported in patients with epilepsy. Equally importantly, it has been shown that the effect of therapy can be correlated with LFP signals both in DBS (Eusebio et al., 2012; Priori et al., 2012) and levodopa therapy (Rossi et al., 2008). In aggregate, these studies suggest that LFP is a promising sensor input for automated systems treating a variety of neurological disorders.

In this work, we describe a platform for investigating these neural signals toward the development of an automated, closed-loop bioelectronic neuromodulation system. The platform comprises tools and a process flow to map the general learning agent to neuromodulation research and enables rapid prototyping of these tools in an implantable neuromodulation device. We use a preclinical, *in vivo* nervous system model to demonstrate the functional components of the system: collection of neural data, identification of relevant features (i.e., biomarkers), development of the algorithm, and consolidation of the algorithm into an implanted device.

## System strategy and infrastructure

**Figure 3:**
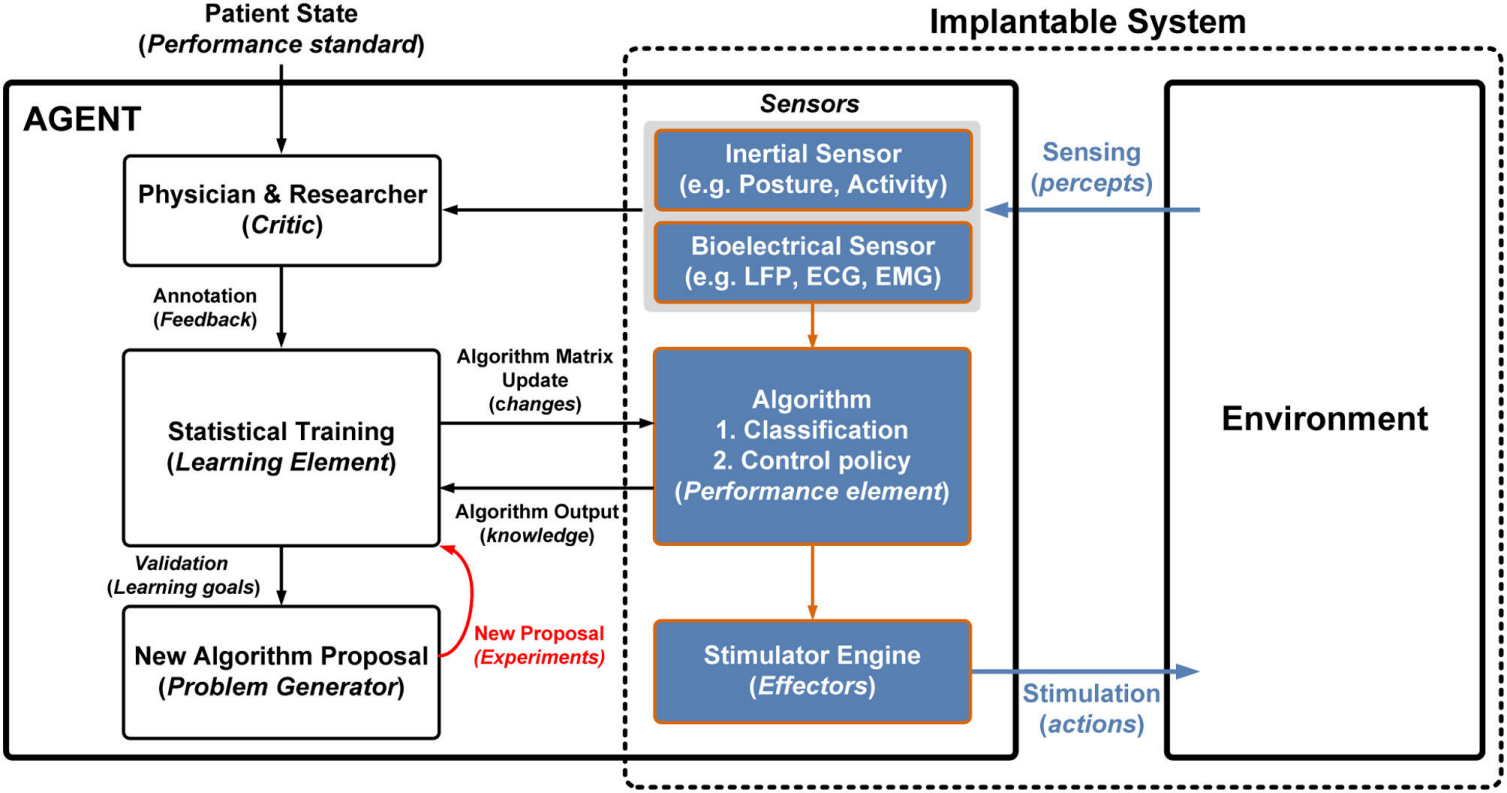
**Mapping a generalized learning model to the neuromodulation system; the components that are implanted are highlighted by the dashed box.** The shaded boxes represent the implanted components that interface with the environment. Algorithm prototyping occurs in the agent where the physician and researcher can generate new algorithms based on historical data and algorithm performance.

To implement this system we mapped the general learning agent functional blocks into the neuromodulation domain (Figure 3). The interface is bi-directional, extracting measures of neural state through percepts and actuating states in the nervous system through effectors. Percepts are received through a combination of sensors that include bioelectrical sensing from electrodes (e.g., ECG, EMG, and LFP) and inertial sensing, (e.g., posture and activity). The effector pathway is defined by electrical stimulation pulse patterns, with parameters similar to approved therapy devices.

The challenge in designing the performance element is that characteristics of both percepts and effectors are still evolving. The algorithm addresses this ambiguity through use of classifier portion that maps sensed signals to estimates of state and a control-policy portion that maps state estimates into a desired stimulation.

**Figure 4:**
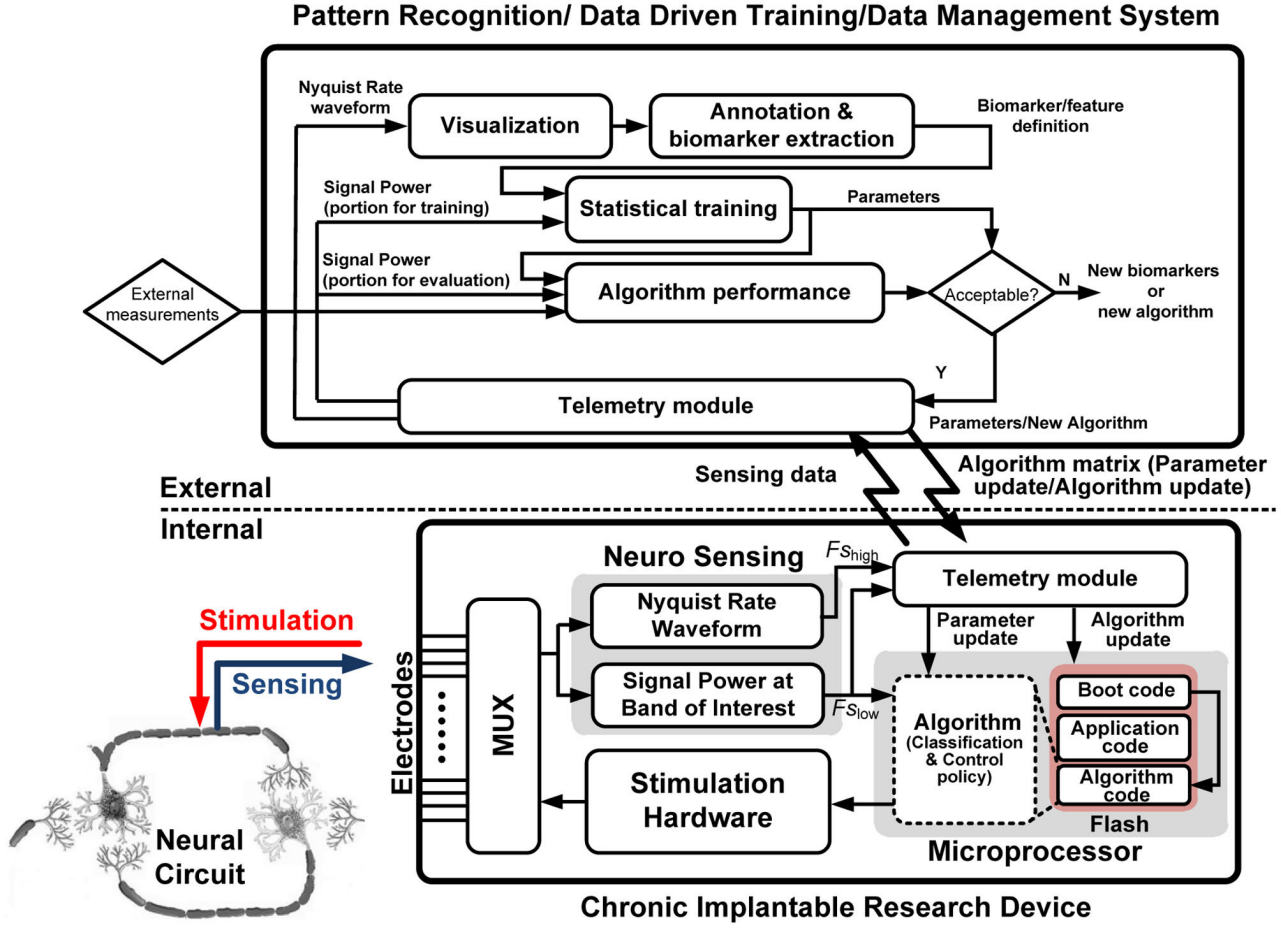
**Functional flow diagram of the hybrid implantable system with the internal and external partitions denoted**.

We have implemented the learning system using an implantable research device and external application tool coupled with real-time telemetry; the system is illustrated in Figure 4. We call this partition of external learning elements that can be transferred to the implantable device performance element a “hybrid” design approach. The goal is to construct a complete platform (combining hardware, software, and firmware) for the learning procedure. The learning protocol includes four main steps from initial exploration to a chronic prototype for validation: collection of sensed neural data; design of the performance element's classifiers based on biomarkers; development of the performance element control policy based on measured neural system identification; and embedding of the performance element into the device for chronic validation.

To do this, we designed a system with the following features:

◦ Implantable device for delivering stimulation including the following components:
  • Bioelectric sensing with 4 bipolar sensing channels with 150 nV/rtHz noise floor without stimulation and 300 nV/rtHz noise floor with stimulation (nb: Stanslaski et al., 2012 describes constraints of sensing during stimulation).
  • Inertial sensing with a custom three-axis accelerometer with a 10 mg-rms resolution floor drawing under 600 nW/axis (Denison et al., 2007).
  • Stimulation using a commercially available neural simulator system with accepted therapy.
  • Embedded algorithm with independently modifiable classifier and control-policy algorithms.
◦ External tool for learning and prototyping classifiers to translate sensor data to state estimates.
  • Save, parse, and annotate data collected from implantable device.
  • Implement, prototype, and compare machine learning algorithms.
  • Develop and test classifiers for the implanted system.
◦ External tool for learning and prototyping control policies to translate state to stimulation updates.
  • Stream data directly from the implantable device to an external processor with latency less than 1 s (0.5 s typical).
  • Send stimulation parameter updates to the implantable device with latency from command to stimulation at the electrode in less than 1 s (0.5 s typical).
  • Monitor state transitions in classifier and control-policy algorithms.
◦ Telemetry system for retrieving data, modifying classifiers, prototyping control policies, and rewriting device firmware.

The key for this system is to integrate all necessary elements to provide a complete platform for an accelerated learning procedure amenable to rapid-prototyping and clinical translation. The details of these steps follow below.

### Collection of sensed neural data

The design of the performance element starts with data collection. While there are many methods to sense biopotential data, fully implanted devices offer the advantage of higher signal fidelity than fully external devices (e.g., EEG), reduced infection risk, and improved chronic, ambulatory data collection capability compared with implanted devices with external components (e.g., externalizing leads during DBS surgery or the Hermes-D system). We have previously described the design and implementation of our fully implanted, bi-directional neural interface (Rouse et al., 2011). In brief, the device contains both sensing and stimulation components. The stimulation feature embodies the capability of a commercial DBS system. Biopotential sensing is enabled with a custom-integrated interface chip that allows for measurements of LFP generated from EMG, ECoG, LFP, and ECG (Avestruz et al., 2008), with noise floor of 150 nV/rtHz without stimulation and 300 nV/rtHz with stimulation (nb: Stanslaski et al., 2012, gives details and constraints of sensing during stimulation). The custom integrated circuit (IC) provides data analysis for up to four bipolar channels, which are selectable between Nyquist-rate waveforms (i.e., time channels) and spectral power at specific frequency bands of interest (i.e., power channels). The time channels provide complete spectral information; however, they incur the penalty of much higher power consumption. Power channels, on the other hand, extract a power envelope that is down-sampled to 5 Hz prior to digital signal processing. The reduction of signal dynamic range prior to digitization is a common technique for saving energy in micropower systems. The design model is to use the time channels for neural system identification, including identifying biomarkers and to transfer to the power channels to optimize efficiency chronically. The inertial element uses a micromachined three-axis accelerometer that transduces capacitive fluctuations to a voltage output. The resolution floor of the inertial element is 10 mg rms, in a 20-Hz band of detection. The sensor draws a total of 2 uW during normal operation, which minimizes longevity impact in the device (Denison et al., 2007). The sensor inputs from bioelectric and inertial sensors can be fused together in the algorithm, if desired.

Data acquisition also provides an opportunity for optimizing efficiency. While the device supports streaming telemetry for time and power channels, it is limited to environments in which the subject is close to a telemetry system, and desired data sampling frequency is low. Event triggered recordings allow for timed segments of high sampling frequency data when the subject is ambulatory. Triggers include user programmable, timer-driven intervals; embedded classifiers; external subject button presses; or combinations thereof. For a typical event structure like motion or seizure onsets, an 8-s loop recording could be applied for two recording channels. With a typical data rate of 422 Hz, approximately 200 recordings can be stored by the embedded SRAM until it needs to be downloaded and cleared. To organize and manage the resulting number of files gathered over a longitudinal study, a file system was developed to provide data structure to researchers. Information such as event time stamps, parameter settings, and event type is embedded in the data during recording and automatically extracted as a companion file to the data. The combination of the custom integrated hardware, signal processing strategy, and data gathering infrastructure facilitates the design of the performance element.

### Learning → performance element I: classification

**Figure 5:**
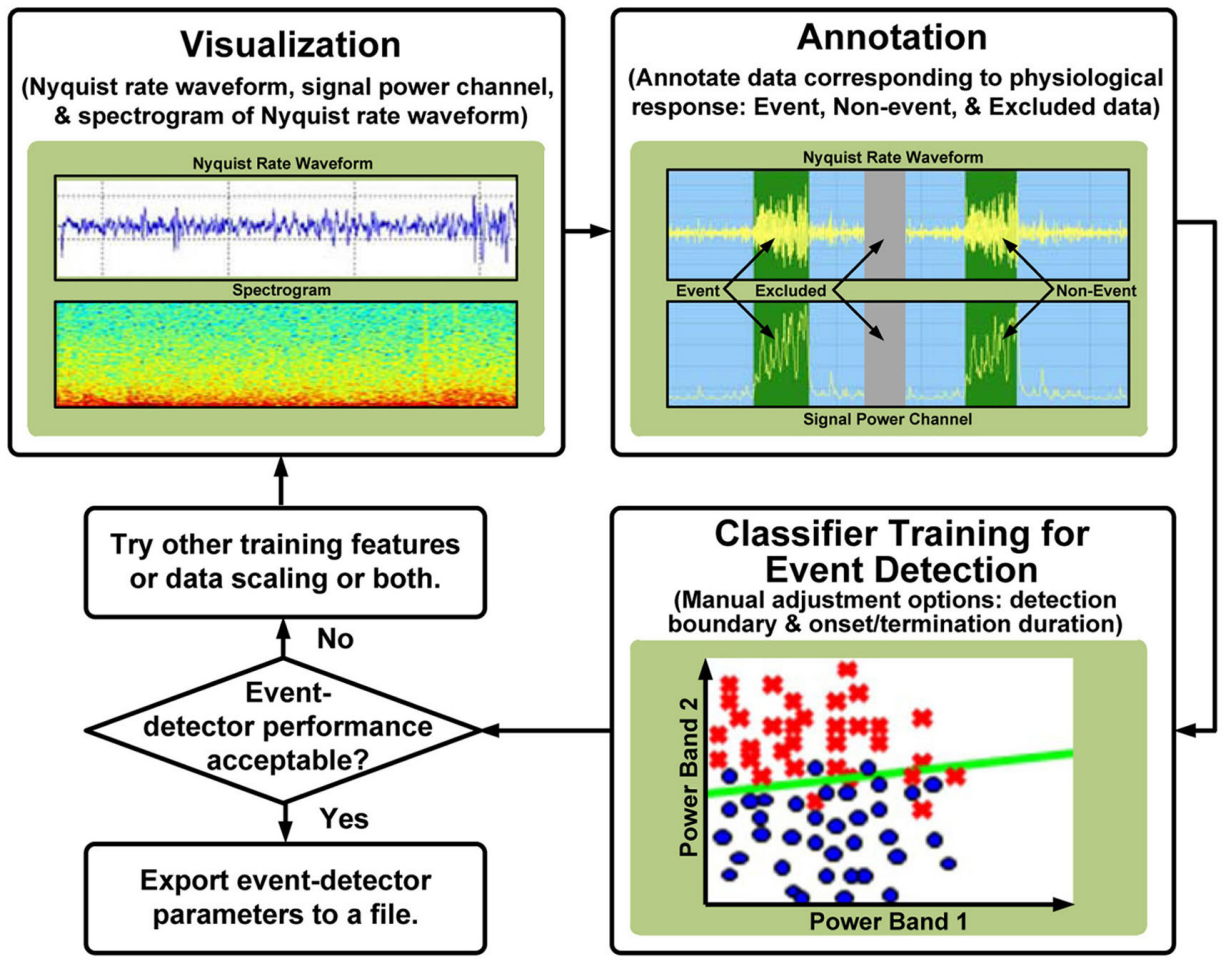
**Functional flow for data annotation and classification using the external software tool**.

The first subsystem of the performance element is a classifier to estimate the state of the nervous system from the sensed LFP biopotentials. Following the hybrid approach of our platform, we implement the classifier as both an internal function of the implantable device and as an external tool for learning and problem generation; the functional flow of the tool is illustrated in Figure 5. The external tool allows users to visualize time domain and spectral data, graphically annotate biomarkers of interest, and automatically generate classifiers using supervised machine-learning algorithms. In addition, classifier sensitivity and specificity can be adjusted manually to obtain the desired performance. The resulting classifiers can be stored and compared using automatically computed detection statistics. Beyond data manipulation, the key value of the tool is its relationship with the implanted device; the tool:

1. Serves as a data repository for grouping and sorting data files from different recording sessions.
2. Parses data collected from the implanted device, automatically accounting for differences in formatting and recording settings.
3. Creates algorithms that can be uploaded directly into the implantable device.

The default on-board classifier algorithm is a linear-discriminant using a modified Fischer-discriminant approach; it is a linear decision boundary in a user-selectable feature space that identifies an event signal sample from other samples. The algorithm was designed using reduced set methods as described in (Shoeb et al., 2009). The use of the multi-dimensional linear boundary was found to optimize trade-offs in power consumption, latency, sensitivity, and specificity. Recent work by Lee describes a similar trade-off calculation and supports our design choices (Lee Kyong et al., 2012). The on-board algorithm can be used for detecting events, which are time-stamped and used to trigger recordings while the subject is ambulatory, thereby reducing current drain nearly 100-fold and reducing classification latency 5-fold, from ~1 s to ~200 ms. If the biomarker's characteristics warrant a more complex classifier or shorter latency, the algorithm can be updated, trading off power consumption.

### Learning → performance element II: control policy

The second algorithm subsystem is the control policy that maps the state estimate into an optimal stimulation sequence. Like the classifier algorithm, we implemented the control-policy algorithm both internal to the device and as an external system for learning and problem generation. Non-linearities in network dynamics heighten the need to sample many input–output pairs for system identification. This can be accomplished in two ways:

First, the external tool may be used to sweep any stimulation parameter (e.g., amplitude or frequency) while the implantable device senses and saves biopotential data to the internal memory. Once retrieved from the device, system identification is performed by measuring the relationship between the stimulation parameters and biopotentials.

Second, the control policy may be adjusted in real-time on a researcher's device using an external device to wirelessly transfer data: sensed data is passed to the researcher's device and control-policy output is passed to the implantable device. This capability enables prototyping algorithms including the use of tapped-delay lines and time synchronizing with other sensors and hardware, and deriving a variety of signal features (e.g., phase amplitude coupling). The external device ensures data integrity in both directions through cyclic-redundancy checks and ensures patient safety by returning the device to safe, pre-programmed stimulation state should the researcher's control policy behave unexpectedly. Additional safety is ensured by allowing the control policy to select only among stimulation parameter boundaries that have been predetermined by the researcher.

For the platform design, particular attention was paid to the latency in the telemetry links, which is a key factor to effectively study the dynamics. In the first generation of development, we required that total latency through the channel be constrained to 1 s or less, and typically under 0.5 s. This degree of latency is suitable for many closed-loop algorithms that operate on timescales of seconds, hours, or days. The inherent latency of the links was dominated by two factors: the first is the data packet format and error correction handshakes using the 175 kHz ISM band, and the second is the internal packet transfer within the bioelectronic device, which, for safety reasons, are secondary interrupt priorities compared to the therapeutic stimulation. Although the latency can be much improved by running in the device, it limits flexibility during the initial learning phase. Therefore, for most cases, the new stimulation parameters are generated externally, where algorithms can be made arbitrarily complex and rapidly evaluated to see if they capture the desired behavior of the neural system. It is highly desirable to validate the behavior prior to committing to verification of embedded firmware due to regulatory constraints and requirements. For example, the platform can implement arbitrary control paradigms such as simple bang–bang controllers (modeled from early cardiac defibrillators) or more sophisticated proportional-integral-derivative and linear-quadratic-Gaussian controllers for achieving the optimal path to the desired state maintenance.

### Committing the performance element to the embedded device for validation

After learning and prototyping the classifier and control policy, the algorithm can be validated by embedding onto the implantable device firmware using telemetry. The firmware uses a dedicated boot loader that allows for a new series of code to be flashed to non-volatile memory inside the device in a few minutes. The firmware in the device is partitioned such that the classifier and control policy can be updated independently of the therapy code, thereby keeping the interaction to that necessary for real-time classification and closed-loop operation. To assist in validation, the firmware is capable of streaming out the classifier and control-policy states in addition to sensed signals in real-time, so that the user has visibility into the algorithm operation. For chronic operation, the state transition information is included in the data log for validation.

## Methods: demonstration of the learning agent architecture

As demonstration of the capabilities of our method and tools, we used the system to investigate, characterize and dynamically modulate the hippocampal dynamics within the circuit of Papez. The circuit of Papez is a thalamo-cortical circuit implicated in temporal lobe epilepsy and involves a reentrant loop involving the hippocampus (HC) and thalamus. The goal was to design from first principles a demonstrative “homeostatic” feedback loop, which would titrate stimulation dynamically to maintain network activity reflected in the field potentials; the intention was to show the capabilities of the technology, as opposed to demonstrate or claim a therapeutic algorithm *per se*. Design of the loop required that we address many issues of neuromodulation design: testing in an awake and freely moving subject, consideration for reliability and repeatability, and chronic implant stability and safety. Methods are detailed from the physiological preparation and technology points of view. The focus of this effort was on exploring the bioelectrical properties of the network and building up a closed-loop system; the conceptual schema for developing inertial-based systems, classifiers and control policies was previously demonstrated with this architecture (Schultz et al., 2012).

### Physiological methods

**Figure 6:**
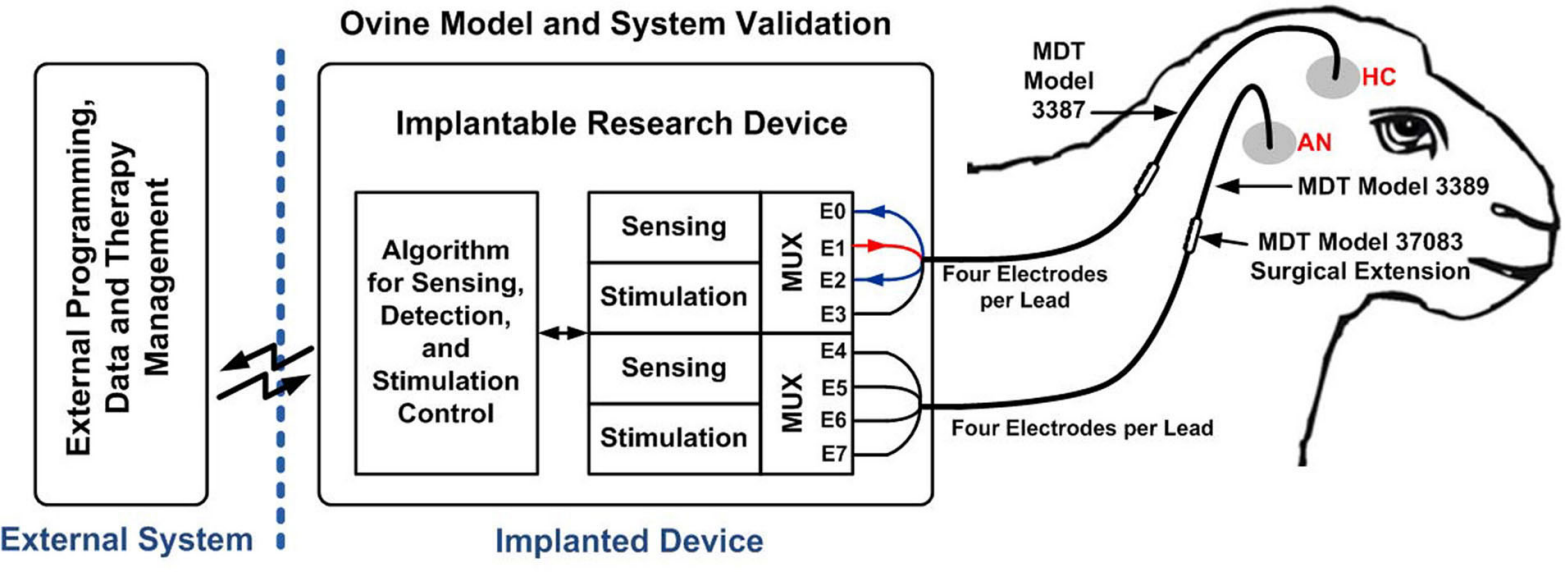
**Closed-loop neuromodulation system implanted in an ovine model.** The figure is reproduced from Stanslaski et al. (2012) with permissions from the IEEE.

The *in vivo* device was chronically implanted in an ovine animal model conducted under an IACUC-approved protocol (Stypulkowski et al., 2011) and is summarized here. Following anesthesia, 1.5T MRIs were collected and transferred to a surgical planning station. Trajectories for a unilateral anterior nucleus (AN) DBS lead (Medtronic model 3389) and unilateral HC lead (Medtronic model 3387) were planned, and leads implanted using a frameless stereotactic system (NexFrame from Medtronic, Inc.). Once lead placement was confirmed based upon electrophysiological measures, Medtronic model 37083 extensions were connected to the DBS leads, tunneled to a post-scapular pocket, and connected to the prototype chronic implantable device. Figure 6 illustrates the overall system placement and setup. Following closure of all incisions, anesthesia was discontinued, and the animal was transferred to surgical recovery.

All sensing and stimulation documented here were conducted in a single, awake sheep resting in a sling. In this particular work, all reported data were recorded from the HC with bipolar montage using contacts surrounding a monopolar stimulation contact (square, biphasic 300 μs pulse width on E1 with far-field return) to mitigate artifacts via common-mode rejection during stimulation (Stanslaski et al., 2012); functional network data from thalamic stimulation and sensing are not shown, but can be found in Stypulkowski et al. (2011). Neural data, stimulus trains and classifier detections were recorded and saved by PC software via wireless telemetry. Data were gathered over 15 months and represents over 18 months of operation with the device completely implanted.

As background to the analysis that follows, our physiological system relies on three qualitatively discernible states in the biological system:

1. *Resting*: defined as the state before any stimulation/neuromodulation has occurred.
2. *After-discharge (AD)*: defined as the state of high-energy LFP, similar to a seizure event, and by characteristic head movements of the subject. In our definition, the AD could occur at any time, independent of stimulation delivery.
3. *Suppression*: defined as the state with activity that is below the nominal resting state.

### Learning flow methodology

The system was deployed on the physiological preparation to develop an embedded closed-loop algorithm using our tools and processes. The technical methods applied the design flow outlined in the system architecture to the physiological preparation:

- **Collection of sensed neural data**
  Using the bi-directional telemetry link and embedded data gathering capabilities, we gathered baseline training data on background network activity. We also used the stimulator and sensing functionality to identify useful biomarkers and understand system transfer functions required for closing the feedback loop.
- **Learning → Performance element I: design of classifier algorithm**
  The software algorithm tool was used to develop classifiers to support the after-discharge detection and verify suppression levels, which were validated using the real-time telemetry link.
- **Learning → Performance element II: development of the control policy**
  After development of the classifiers, the auto-detection of after-discharges and therapy titration was validated using off-line, real-time processing with the bi-directional telemetry link. Key parameters were verified to be acceptable for timing latency. An additional algorithm (data not shown) was tested to show the system could automatically search the parameter space to find acceptable suppression behavior.
- **Committing the performance element to the embedded device for validation**
  The final embedded algorithm implemented three sub-algorithms into a single-state machine: AD detection and mitigation; suppression detector; and parameter search. The code was then downloaded to the device through wireless telemetry, error checked for complete flash writes, and the implant was then activated with the closed-loop algorithm. All states were exercised in the algorithm routine to validate operation. State transitions were also recorded in the device data records for automated annotation of files, allowing for observational validation and algorithm refinement.

## Results

### Collection of sensed neural data: identification of biomarkers and algorithms

We aimed to explore the states of the system to find relevant control-variable biomarkers *in vivo*. Analysis of the post-stimulation data showed decreasing mean beta band power with increasing stimulation amplitude, suggesting suppression of activity, at least locally to the HC (Figure 8, right). To determine whether the network was truly suppressed, we performed a second series of transfer function experiments which measured the pre-stimulation baseline beta band power level followed with a high amplitude delivery (≥1.50 V) “probe pulse” capable of inducing AD. Because our experimental setup was not a seizure model, we used the post-stimulation AD duration as the desired output for assessment of network effect. Through spectral analysis of the data, we observed a potential control variable in the 20 ± 2.5 Hz band (approximately the beta band) that seemed correlated to the qualitatively observed states:

1. Resting state corresponded to relatively constant beta band power (approximately 2.7 uVrms).
2. AD state corresponded to increased beta band power (approximately 30 uVrms).
3. Suppression state corresponded to decreased beta band power (approximately 1.5 uVrms).

**Figure 7:**
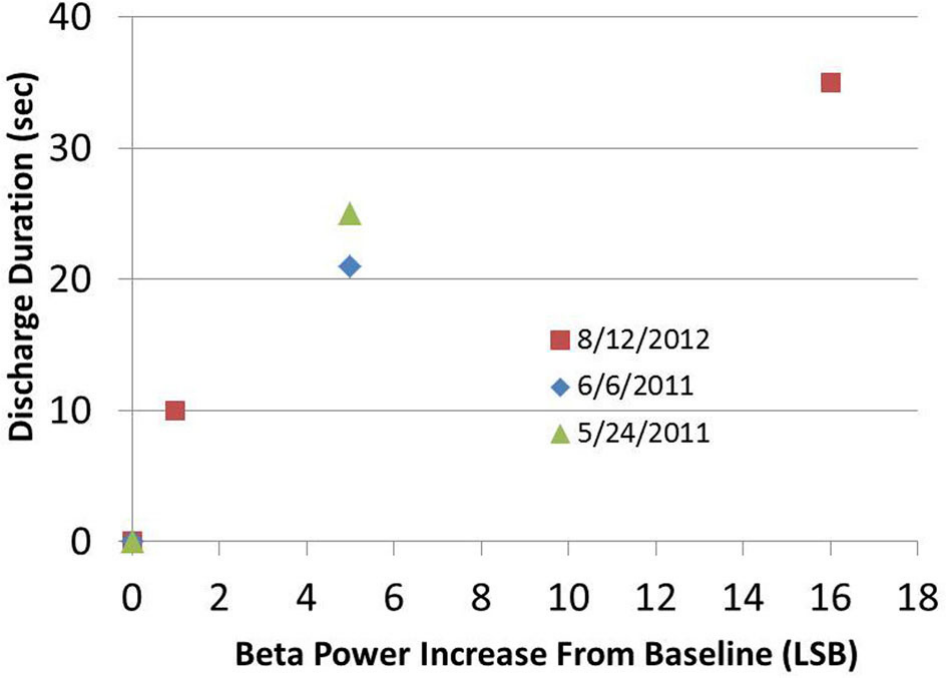
**After-discharge duration as a function of beta band power increase from suppressed baseline.** High amplitude stimulation parameters were kept constant in a given session and were always determined to be sufficient to initiate an AD.

We characterized the biomarker over 15 months of data collection. For data shown, the units of spectral power in all data figures are (uV/rtHz)^2^, with an arbitrary scale referred to as least significant bit (LSB). Results showed that AD generation was a probabilistic function of stimulation amplitude; stimulation below 1.5 V did not result in any AD, stimulation between 1.5 and 1.7 V resulted in occasional ADs, and stimulation above 1.7 V always resulted in ADs (data not shown). Furthermore, AD duration appeared to be a function of the beta band pre-stimulation state; the greater the pre-stimulation beta band power above the defined suppressed state, the greater the AD duration (Figure 7). Furthermore, these observations were robust: the suppressed state beta band power varied by less than 2LSB over the entire duration of the experiment. These results imply that spectral beta band power could be a control variable of interest when modulating network state.

**Figure 8:**
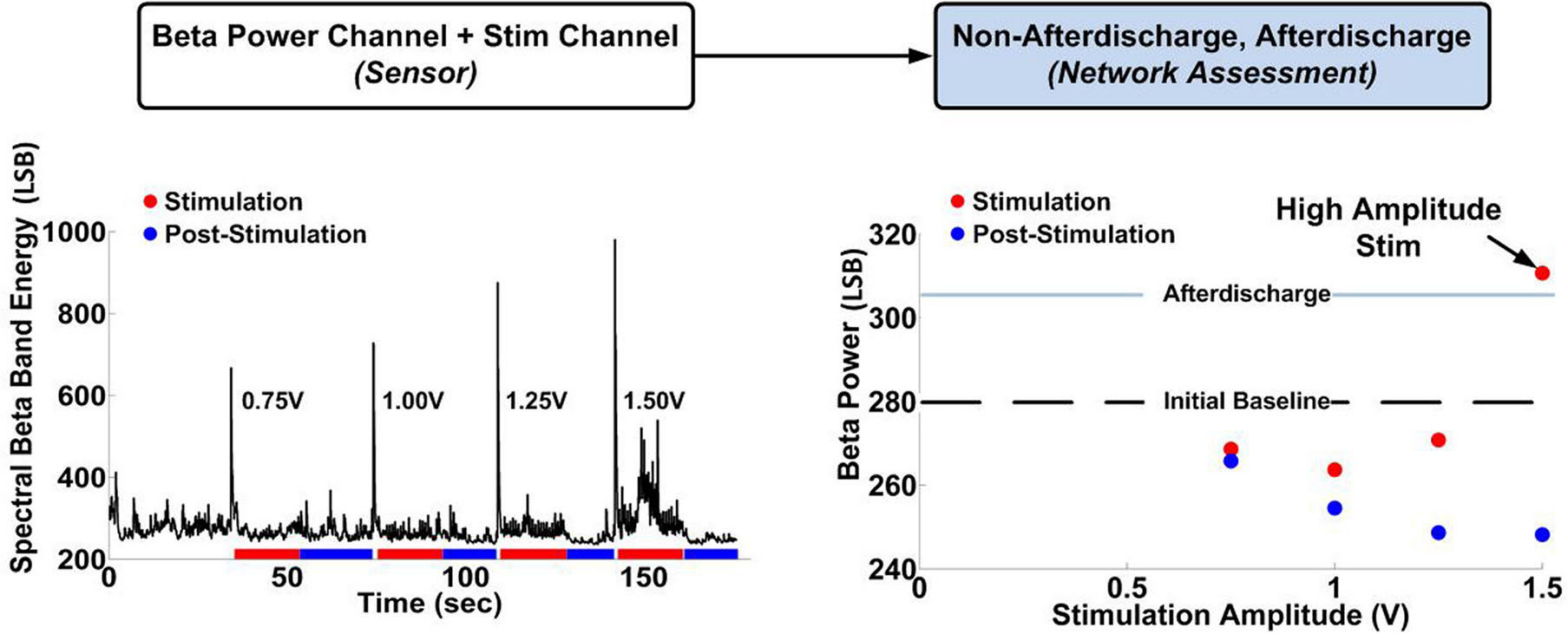
**Determination of the hippocampal network transfer function between stimulation and beta band spectral power.** There is an initial reduction in beta band power at low stimulation amplitudes, followed by an increase in beta band power at higher stimulation amplitudes, resulting in occasional AD during stimulation at 1.5 V.

To further understand the dynamic state of the system, we aimed to characterize the transfer function between our proposed biomarker—spectral beta band power—and stimulation patterns. To characterize the response of the biomarker to stimulation, we ran several titration sweeps. The recorded biomarker signals were captured at rest, during AD events, and during delivery of stimulation at several amplitudes (0.75–1.7 V) and frequencies (50, 120 Hz) in order to determine a reference value to discriminate both suppression and ADs. Figure 8 shows the network response during stimulation (25 s, red) and between stimulation periods (25 s, blue). Importantly, the detection of AD induction required sensing neural activity in the presence of stimulation (Stanslaski et al., 2012) and would have been lost if channel blanking were employed.

The titration sweep for determining network-state response to stimulation is a critical step in designing the neural control algorithm. The data suggest that stimulation can have different effects on the network: while low and moderate stimulation amplitude appears to suppress the network excitability, high stimulation amplitude can induce an AD. Based on these results, we wanted to use our platform to implement a performance element to have two key features: (1) change stimulation amplitude to keep the network at the balance point of suppression and induction of AD and (2) due to the probabilistic nature of AD induction, allow for the detection of AD in real-time to abort stimulation and adjust the stimulation levels lower. To do this, we designed the performance element in two parts: states classification and control policy implementation.

### Learning performance element I: design of classifier algorithms

**Figure 9:**
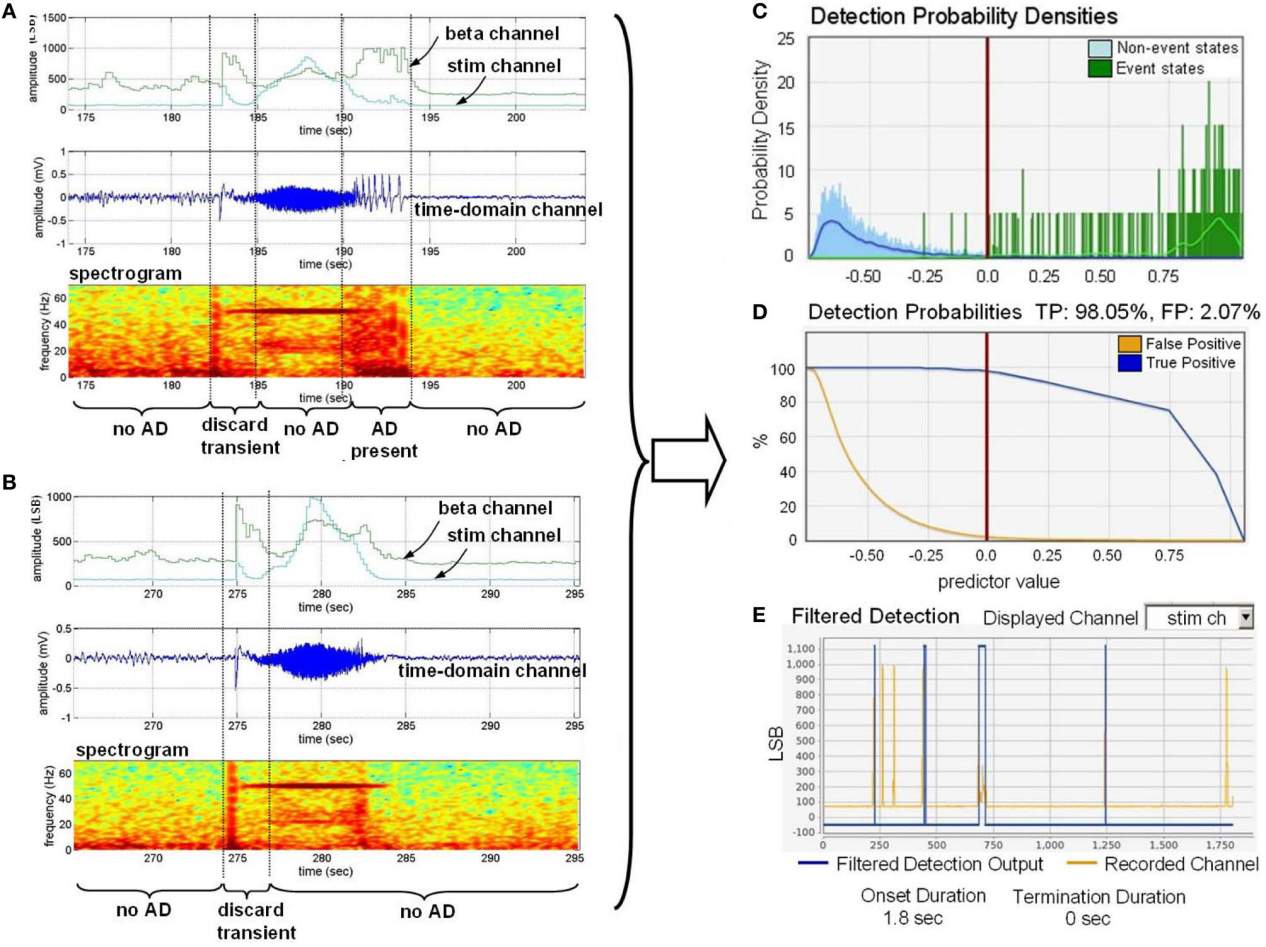
**Training the classifier to detect the onset of after-discharges in the presence of stimulation. (A)** Is a dataset with a representative AD. “Discard transient” periods refer to portions of the signal that were not used in training. **(B)** Is a dataset for providing stimulation artifact without an AD present. **(C)** Provides the histogram of detection states versus distance from the classifier boundary. **(D)** Estimates the true positive (TP) and false positive (FP) percentages based on the classifier. **(E)** Shows the impact of onset and termination constraint logic on detector specificity by overlaying the estimated detector state with recorded data files.

To automate a control loop, we used the observed qualitative correlations with a quantitative algorithm to detect the AD in real-time with a classifier constructed with the external classifier tool. To help mitigate stimulation artifact, we also used spectral band (approximately 70 Hz) to capture stimulation energy in the network without being confounded by observable changes in neural physiology. To achieve this, we applied a measure of stimulation artifact as a feature input within the algorithm to distinguish stimulation result and non-stimulation result as described in Stanslaski et al. (2012). We include the two power channel outputs in Figure 9 for demonstration purposes, showing correlation between the amplitude of beta band power and AD in Figures 9A,B.

After annotation was supplied to the training data sets, we used the tool to develop a linear, binary classifier to detect AD with and without stimulation. The detection probability density plot, receiver operating characteristic (ROC) curves, and detection cross-validation result, which are directly generated by the software tool, are presented in Figures 9C,D, and E, respectively. The detection probability histogram (C) represents the magnitude of the state from the boundary, allowing for multiple dimensions of data to collapse to a single graph biomarker separation. The detection probabilities graph (D) provides an estimate of the true-positive and false-positive rates based on the derived classifier. The filtered detection summary graph (E) allows for the user to set onset and termination duration constraints (i.e., a minimum duration in a classified state before detection is determined) to help improve specificity at the expense of classifier latency. Graph (E) shows an overlay of the classification state over the data. We downloaded and embedded into the implanted device the classifier that optimized sensitivity, specificity, and latency trade-offs.

In addition, we used the tool to develop a separate classifier that could detect the presence of the suppression state based on the beta signal. This was also tested and similarly embedded in the implanted device. Thus, with these classifiers, the state of the neural system could be quantitatively classified on-line as suppression, AD, or resting.

### Learning performance element II: development of the control policy

**Figure 10:**
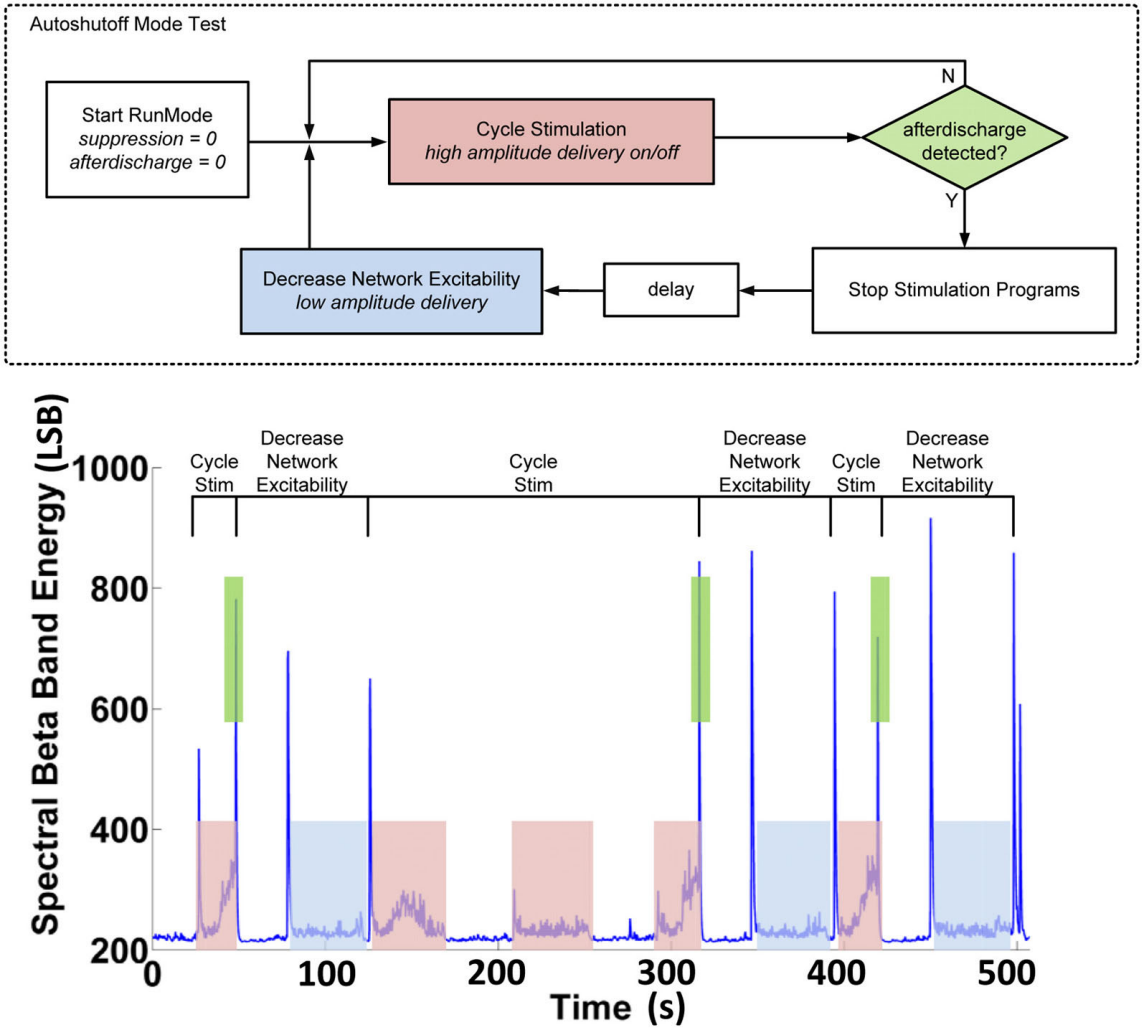
**Hybrid system validation of the auto-shutoff algorithm for preventing sustained after-discharges in the hippocampus**.

With the classifier in place, we next determined the control policy. Given the unknown neural dynamic requirements and algorithm parameters, the control policy was first prototyped using the hybrid development partition to determine the stimulation amplitudes and changes that would be used for each state. Figure 10 illustrates an example of this testing to show that stimulation can induce both the AD state and the suppression state. In this test, the controller logic uses two stimulation programs. In the cycle stim (CS) program, high amplitude stimulation (1.50 V) capable of inducing an AD is cycled on and off, while spectral power in critical bands and classifier state is continuously telemetered out of the device. If the classifier does not detect an AD, stimulation continues to cycle. When the AD state is detected, stimulation is stopped and an alternate setting is applied. The decreased network excitability (DNE) program delivers a lower stimulation level (1.25 V) after a programmed delay for one cycle, then returns to the CS program.

Figure 10 (bottom) shows typical results achieved with the hybrid algorithm. We ensured no false-positive detections occurred in both open-loop and closed-loop cases by examining the time-domain data. Our results demonstrate that open-loop stimulation leads to sustained ADs *post-stimulation* roughly 50% of the time when the cycle stimulation is applied without the algorithm enabled, whereas with the algorithm enabled, the sustained AD probability drops to 0% [*N* = 12, three monitor sessions, 15 months].

### Committing the performance element to the embedded device for validation

As a final prototyping phase, we desired a system capable of embedded operation to enable chronic, ambulatory data collection for long-term validation as well as improved response latency compared to subjects or other observers (e.g., researches, caregivers).

**Figure 11:**
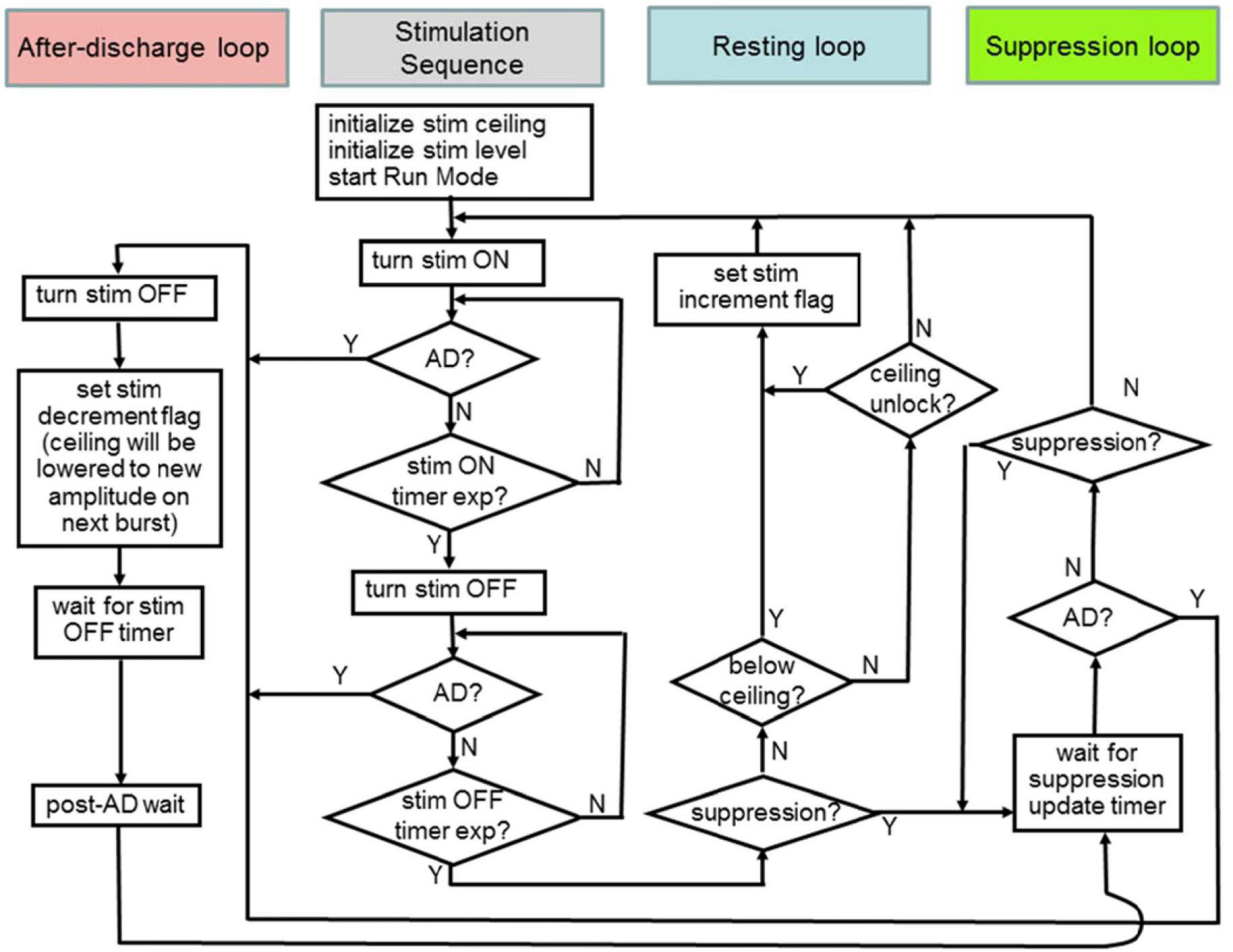
**The embedded control policy for modulating hippocampal network dynamics.** Color codes at the top will be used to mark states in the resulting data summary.

Note: Additional parameters such as initialization variables and counters are also programmable through telemetry and could be refined as needed.

Based on findings with the hybrid system, the device was enabled to run a multi-branch algorithm for hippocampal network dynamics. The algorithms developed for the embedded detector were merged into a common state machine. As shown in Figure 11, this included the three critical loops for the algorithm corresponding to the states of the system, all of which share a common *stimulation sequence* forward loop. The beta band power threshold for determining the state classification was determined using the classifier. In addition, we prescribed an increment of 0.05 V and decrement of 0.1 V for stimulation controllers—i.e., slow attack, fast recovery for attempting to maximize safe searches of the parameter space.

- *Suppression loop*—detects suppression after stimulation and maintains defined suppression in the HC based on network activity within a broad beta band (10–30 Hz); the detector gates when stimulation pulses would occur based on measured spectral power.
- *After-discharge loop*—detects after-discharge and aborts stimulation, decrements stimulation amplitude, and sets a new “ceiling” on the stimulation level for future excitation patterns to avoid future AD events.
- *Resting loop*—detects resting state and increments stimulation amplitude to verify the ceiling is still valid; this loop is activated when suppression is no longer being achieved with the suppression loop to counteract slowly changing behavior such as circadian patterns, medication dosing, etc.

The algorithm firmware was downloaded into the device and validated with cyclic-redundancy checking.

**Figure 12:**
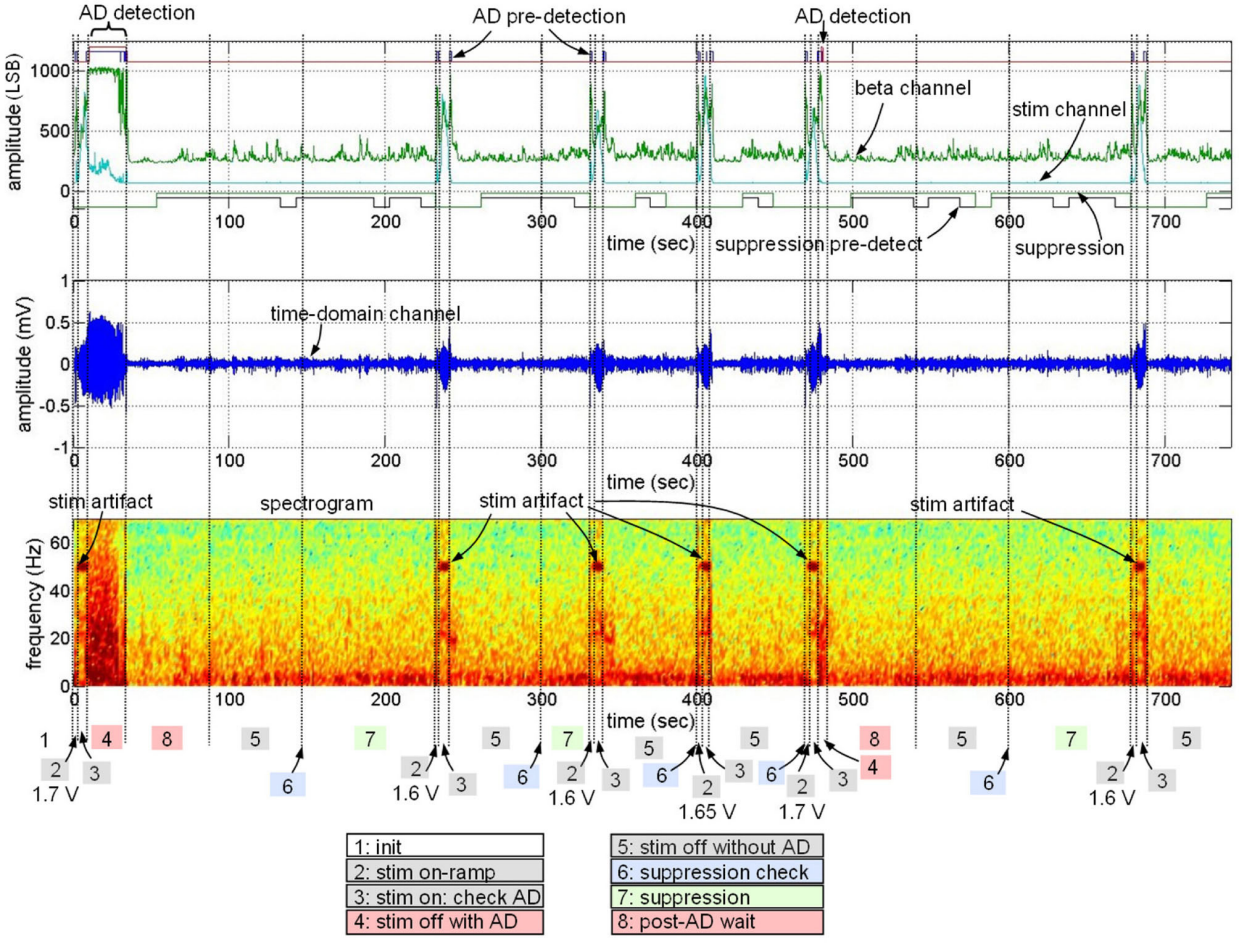
**Data sample from embedded algorithm (Figure 11).** The sample demonstrates data associated with detection of seizure-like events in the presence and absence of stimulation and change stimulation parameters, resulting in no observed after-discharges. “Pre-detection” refers to the period of time when the onset or termination constraint has not yet been met.

The embedded algorithm was then evaluated with on-line processing in the ovine model. Figure 12 presents a typical outcome of the standalone implantable device with the algorithm embedded; we demonstrate all possible states of the of the control policy in this data sample. We start by stimulating at an amplitude known to generate AD, resulting in appropriate stimulation shut-off. Then, stimulation is ON with reduced stimulation amplitude (from 1.7 to 1.6 V). Stimulation at this level produces suppression for one cycle, leading to maintenance of this stimulation level for 1 cycle. On the next cycle, however, suppression is not detected, resulting in stimulation increase to 1.65 V and then again to 1.7 V. At 480 s, the 1.7 V stimulation again leads to an AD. The stimulation is again turned off due to the AD detection and the stimulation level is returned to 1.6 V. This testing showed that the learning procedure could result in a fully embedded solution, from initial identification of biomarkers and transfer functions to a fully-embedded control policy operating *in vivo*.

Several practical points are also worth noting. First, the algorithm is power efficient, because it runs reliably with total current drain less than 20 μW with the addition of sensing and algorithm control. This represents roughly 10% of the nominal therapy power used in movement disorder neuromodulation system. Second, the algorithm shows robustness because signal power channel baseline is stable over 15 months with variation within 2 LSB, which is more than 20 times smaller than the AD detection threshold. Finally, the control policy is restricted to a bounded set of stimulation parameters with programmable inter-locks, thereby helping to ensure tolerability and safety.

## Discussion

Automated closed-loop control systems may potentially improve neuromodulation therapies by reducing latency for therapy adjustments and personalizing therapies to improve patient health. These approaches rely on improved understanding of the nervous system dynamics and how they drive the mechanisms of action for neuromodulation. Mapping these concepts to a learning agent framework helps define key components that can lead to better characterization of the system: sensors for chronically collecting data; effectors for modulating the network; and algorithms for translating data into stimulation parameters. The investigational platform described here fills a gap in current technology by enabling a process methodology for designing and prototyping these algorithms and embedding them in an automated closed-loop neuromodulation device.

In this work, we demonstrated a platform consisting of an implantable device integrated with external tools for developing classifier and control-policy algorithms. We tested the platform in a system that exhibited contrasting behavior with respect to stimulation amplitude, motivating our algorithm design to find the fine balance point between over- and under stimulation. One of our significant findings was a potentially non-monotonic relationship between stimulation amplitude and system response: beta band power was reduced from baseline at low stimulation amplitudes, while it was increased at higher stimulation amplitudes, resulting in occasional AD. These results imply that neural feedback may be an important consideration in determining the optimal stimulation amplitude.

While we performed our experiments in an *in vivo* ovine, our investigational approach could be applied to the study of other disease states, such as Parkinson's disease, essential tremor, epilepsy, or other neurological conditions. Preliminary exploration of the automated algorithm supports the design of other closed-loop systems using similar control policies to those described here (Eusebio and Brown, 2009; Priori et al., 2012). Furthermore, our system is not limited to neural biopotentials; we can theoretically record any biopotential of sufficient amplitude (e.g., EMG). These biopotentials, along with other sensor data, may be useful in prototyping and validating algorithms for future automated closed-loop systems (Yamamoto et al., 2012).

Our design involved several practical considerations. Perhaps most importantly, we designed the generalized learning system on a chassis that has received prior approval for select therapies. Building off an established foundation helps to lower the translational barriers to exploring advanced systems. An additional key design element is the ability to sense activity in the presence of stimulation (also described in Priori et al., 2012). Our results demonstrate the potential importance of network phenomena that occur while the network is being modulated—especially while characterizing transfer functions of the nervous system that might underlie mechanisms of action. In this work, this capability allowed us to monitor for evidence of AD during the stimulation as well as dynamically adjusting the stimulation ceiling as a function of suppression state. These phenomena may be missed by neural sensing architectures that blank out the signal chain during the stimulation (Sun et al., 2008).

Another practical consideration is that the learning pathway is amenable to chronic embedded algorithm operation, particularly in light of the trade-offs between complexity and performance versus simplicity and power consumption (Lee Kyong et al., 2012). The offline analysis and hybrid design approach allow for rapid prototyping of concepts before commitment to embedded firmware. Once embedded, the power draw with our system could be reduced to 20 uW, below 10% of existing nominal therapy power for Parkinson's disease, and latency can be reduced to approximately 200 ms. In the future, use of complementary sensors such as accelerometers and patient feedback may enable algorithms to maintain simplicity and efficiency without sacrificing performance. Ultimately, the ability to titrate stimulation to therapy using responsive algorithms (such as the suppression loop) could potentially yield a net energy savings of chronic responsive systems.

Finally, the experiments allowed us to observe overall reliability of the system. Observed signals of network states were stable over the course of the 15-month experiment, providing evidence of robustness in our detection algorithms (>20-fold margin) to detect state changes. This finding, combined with other results (Stypulkowski et al., 2011), provides initial confidence in the reliability of the system in an *in vivo* environment. In addition, our control-policy implementation used bounded stimulation parameters to ensure tolerability and safety. The chronic reliability and means of ensuring safety provide both a mechanism for longitudinal learning to occur within one subject and chronic validation of the methods, thereby greatly increasing the likelihood of clinical translation.

The study does suffer from limitations, mostly tied to the choice of animal model used for validation. First, the validation is tied to physiology measures and not a true disease model. The ultimate therapeutic utility of the algorithm will require additional testing in animal and clinical models which might drive refinement of the algorithm. In addition, the hybrid system is limited by telemetry latency. Future investigations characterizing the latency of the feedback loop may be needed to better understand this impact vis a vis neural dynamics. System latency may be particularly relevant when stimulating multiple neural regions, such as in stimulating pairs of neural targets or in functional electrical stimulation of muscle in response to sensed neural signals. Ultimately this latency is addressed when embedded in the system, but might limit the broader application of the hybrid design process.

In summary, we believe increased understanding of the nervous system with such platform systems may lead to improved technical capability to modulate the nervous system to address pathophysiology. As these systems mature, they can be embedded into devices to augment and potentially correct for a malfunctioning nervous system.

### Conflict of interest statement

The authors are all employees of Medtronic; Paul Stypulkowski, Scott Stanslaski, Randy Jensen, David Carlson, and Tim Denison are also Stockholders in Medtronic.
